# Marginalized voices on HPV vaccination in India: exploring knowledge, attitudes, and acceptance

**DOI:** 10.3389/fgwh.2026.1791203

**Published:** 2026-04-22

**Authors:** Uma Vasudevan, Angela Kelly-Hanku, Priya Limbu, Paula Jops, Ranjitha S. Shetty, Ekta Gupta, K. Eshwari, Shalini Singh, Andrew Vallely, Preety R. Rajbangshi

**Affiliations:** 1 Department of Public Health, Amrita Institute of Medical Sciences, Amrita Vishwa Vidyapeetham, Kochi, India; 2 The George Institute for Global Health, New Delhi, India; 3 Kirby Institute, The University of New South Wales (UNSW), Sydney, NSW, Australia; 4 Department of Community Medicine, Kasturba Medical College, Manipal Academy of Higher Education, Manipal, India; 5 ICMR-National Institute of Cancer Prevention and Research, Noida, India

**Keywords:** cervical cancer prevention, gender, human papillomavirus (HPV) vaccination, marginalized communities, qualitative research, vaccine acceptability

## Abstract

**Background:**

Cervical cancer remains a major cause of morbidity and mortality among women in India, particularly among marginalized communities who face heightened vulnerability to Human papillomavirus (HPV)-related disease. As India prepares for the national rollout of HPV vaccination for girls aged 9–14 years, understanding community-level knowledge, attitudes, and acceptability is essential to ensuring equitable uptake. This study explored perceptions of HPV vaccination among marginalized populations across three diverse Indian settings.

**Methods:**

A multi-site qualitative study was conducted between February and October 2023 in Delhi-NCR, Tripura, and Karnataka. Semi-structured interviews (*n* = 89) and gender-specific focus group discussions (*n* = 12) were undertaken with tribal women, migrants and internally displaced women, women living with HIV, female sex workers, and men from tribal and migrant communities. Data were analyzed thematically using Braun and Clarke's framework.

**Results:**

Awareness of HPV vaccination was extremely low, with only 14 participants having heard of the vaccine, and only one male participant aware of it. While many participants expressed willingness to vaccinate themselves or their daughters, others held cautious or negative views rooted in concerns about side effects, fertility, moral beliefs, and confusion linked to the COVID-19 vaccination experience. Gendered decision-making norms shaped vaccine acceptance, with many women relying on husbands or senior family members for consent. Participants emphasized culturally grounded, community-based strategies for vaccine introduction, including locally tailored communication and engagement through trusted community actors.

**Discussion and conclusion:**

This multi-site qualitative study highlights the interplay of limited knowledge, gendered power dynamics, stigma, and trust in shaping HPV vaccine acceptability among marginalized populations in India. To enhance vaccine knowledge and acceptability, tailored community engagement, culturally responsive communication, and inclusive decision-making processes are essential. As India advances towards national HPV vaccine rollout, addressing these factors will be critical for achieving equitable coverage and supporting cervical cancer elimination goals.

## 1 Introduction

Human papillomavirus (HPV), which is vaccine preventable, is the leading cause of cervical cancer (1). It is one of the most common sexually transmitted infections among women globally, primarily spread through sexual contact, including vaginal, anal, and oral sex (2, 3). Persistent infection with high-risk sub-types 16 and 18 accounts for 70% of all cervical cancer cases, making it the leading cause of the disease (4). The impact of HPV-related cancer is especially severe in low- and middle-income countries (LMIC) including India, where limited access to HPV vaccination and cervical screening contribute to ongoing infection, disease, and late diagnosis with poor outcomes (5–8). In India, cervical cancer is the second leading cause of cancer mortality among women (9–11), highlighting the urgent need to both improve timely diagnosis and treatment, but also ensure Indian girls are protected against HPV infection.

In 2020, the World Health Organization (WHO) launched the “Global Strategy to Accelerate the Elimination of Cervical Cancer” as a public health problem by 2030. This initiative emphasizes a comprehensive approach to elimination that is centred on three pillars: vaccination, screening and treatment for cervical cancer. WHO has set the ambitious target of 90% of girls to be fully vaccinated against HPV (5). Vaccination to prevent HPV infection is the most effective and simplest strategy to prevent cervical cancer, especially when administered to girls before exposure to the virus (12).

HPV vaccination is now an emerging priority in India. Following the licensing of bivalent and quadrivalent vaccines in 2008, India initiated limited HPV vaccination programs in the states of Andhra Pradesh and Gujarat. A major milestone was achieved a decade later with the successful state-wide rollout in Sikkim during 2018–2019, which played a pivotal role in shaping the country's national HPV vaccination strategy (13). In 2022, India launched its first locally-produced quadrivalent HPV vaccine (14), representing a significant advancement in cervical cancer prevention and paving the way for potential inclusion in the nation's universal immunization programme, consistent with the WHO's global elimination strategy (15). The Ministry of Health and Family Welfare, Government of India, is currently planning a national roll out of universal HPV immunization for girls aged 9–14 years (16).

The success of a country's HPV vaccination program is highly dependent not just on reliable supplies and availability of the vaccine and the human resources needed to administer it, but is critically influenced by the attitudes, knowledge and discourses that surround the vaccine, including narratives of morality and adverse events, that lead to community acceptance or rejection of vaccination (17–20). Public narratives, particularly those in the news and other media outlets, have been shown to be significant sources for the spread of misinformation, and can actively undermine public confidence in vaccination and as a result, affect vaccination uptake (18–21). Issues of acceptability of the HPV vaccine (and its health communication messaging) are important, not only during implementation, but are critical while incorporating the HPV vaccine into routine immunization programs, or to do so at scale (22, 23).

The same challenges identified in other LMIC settings, including socio-cultural norms, and health communication and literacy, also affect the uptake of preventive healthcare measures in India, notably in the area of routine immunization (24, 25). Against this backdrop, the government of India has stated that it plans to introduce the HPV vaccine in the national immunization programme soon, but as yet, no official date has been given. In anticipation of this, locally relevant sociocultural research on HPV vaccine acceptability and delivery is needed to inform the national program, notably amongst marginalized women and girls. The research on HPV vaccine acceptability and uptake in India to date, though emerging, remains relatively limited to the general population or specific groups such as health workers or students (26–30). With such diverse populations, and many marginalized communities, India's success will depend on ensuring that it addresses the needs among marginalized populations who have little or no knowledge about cervical cancer and limited access to health care services. Such women, including female sex workers, women living with HIV, indigenous women, migrants and internally displaced populations face unique challenges. These women are known to have a higher risk of developing cervical pre-cancer and cancer, especially those living with HIV (31–33).

Such women often report additional barriers to accessing health services, including cancer prevention programs. Barriers include poverty (34–36), social isolation (37–39), mistrust of healthcare services (40, 41), having culturally and linguistically specific needs that mainstream services often fail to meet (42–45), limited health awareness (46, 47), and experiences of stigma associated with sexually transmitted infections (STI) (48–50). These challenges make women from marginalized communities, disproportionately disadvantaged and at higher risk of discrimination.

This paper adds to the growing interest in HPV vaccination in India as well as other LMIC, focusing on marginalized populations. Based on a qualitative study in three Indian sites, it explores knowledge, attitudes, and acceptability of HPV vaccination for cervical cancer prevention. The paper highlights barriers to vaccine uptake and provides evidence-based recommendations to improve awareness and acceptance among India's most marginalized communities.

## 2 Materials and methods

As part of a larger multi-site qualitative study, we included tribal women, migrant and internally displaced women, women living with HIV, and female sex workers to understand socio-ecological factors influencing their cervical cancer prevention, screening, and treatment.

**Figure 1:**
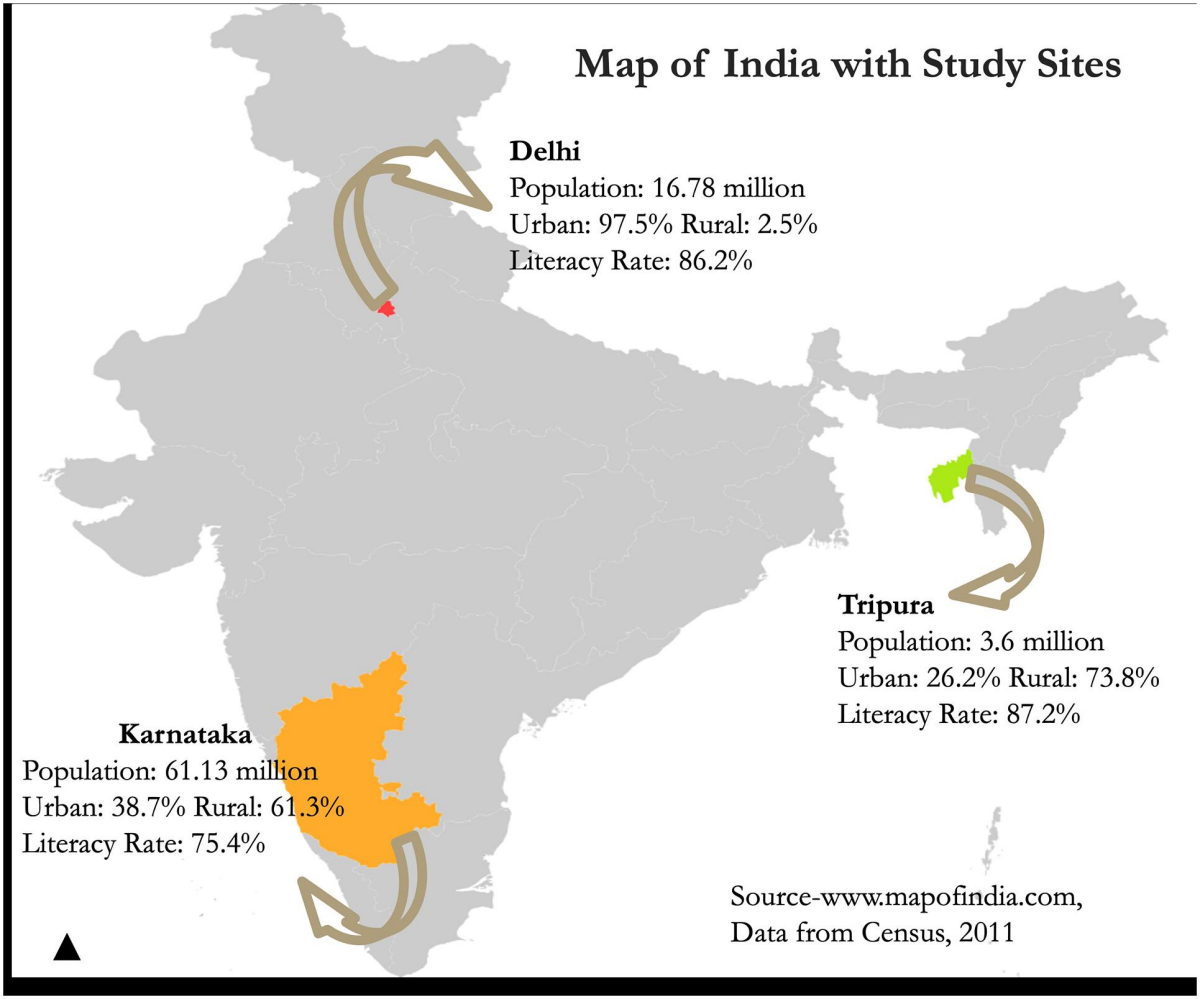
Map of India with study sites.

We conducted semi-structured interviews and focus group discussions with women and men from these communities in three geographically and demographically diverse locations: Delhi-National Capital Region (NCR), Tripura, and Karnataka (Figure 1).

### 2.1 Research setting

NCR comprises Delhi and adjacent urban and peri-urban districts in neighboring states, characterized by internal migration, socio-economic disparities, and a mix of public and private health services (51, 52).

Tripura, a small rural state with a significant tribal population, hosts internally displaced populations (IDPs) affected by ethnic conflict. Despite relatively well-utilized public health services, geographic isolation and weak infrastructure limit care in remote areas (53, 54).

Udupi district in Karnataka represents a coastal rural/semi-urban setting with a mixed economy and relatively strong health infrastructure (55).

#### 2.1.1 Participant recruitment

Women aged 30–65 years, targeted for cervical cancer screening as per national guidelines (56) were included. Men from tribal, migrant, and internally displaced communities were also included for gender insights.

Participants were recruited through various strategies. Women who had screened at health facilities were identified through outpatient registers and contacted by the health facilities. Women who hadn't screened were recruited via community health workers using snowball sampling. Men were recruited through community health workers using snowball sampling.

Study participants included female sex workers, women living with HIV, internal migrants facing health access barriers from Delhi; IDPs with disrupted livelihoods, social networks, and healthcare access from Tripura; and HIV-positive women, tribal populations, and seasonal migrants from Udupi.

### 2.2 Data collection

We conducted field-based data collection from February to October 2023, involving 89 semi-structured interviews (SSI) with women and 12 focus group discussions (FGD) with men and women separately (see Table 1). Interviews were conducted with screened (*n* = 34) and unscreened women (*n* = 55). FGDs included 8 with women and 4 with men across the three sites, with homogeneous groups formed based on community (type—for women: tribal, women living with HIV, migrants, internally displaced communities, and female sex workers; for men: tribal, migrants, and internally displaced communities).

**Table 1:** Summary of semi-structured interviews (SSI) and focus group discussions (FGD) conducted.

Data collection methods	Category	Sites			
		Delhi	Karnataka	Tripura	Total
Semi-structured interviews	Women screened	14	19	1	34
	Women Unscreened	17	29	9	55
	Total	31	48	10	89
Focus group discussions	Women (*n*)	3 (26)	3 (16)	2 (15)	8 (67)
	Men (*n*)	1 (6)	1 (8)	2 (14)	4 (28)
	Total (*n*)	4 (32)	4 (24)	4 (29)	12 (85)

Before each interview and group discussions, consent from each participant was taken to conduct and record the discussion. If any participant had a query the research team addressed, it before starting the interview. All interviews and FGDs were digitally recorded, lasting 30–45 and 60–90 min, respectively.

Semi-structured Interview guides explored participants' knowledge, attitudes, and practices regarding cervical cancer prevention, screening, and treatment, including barriers and facilitators to accessing services. They also covered HPV vaccination knowledge, attitudes, and acceptability. FGD guides complemented SSIs, generating collective perspectives on cervical cancer prevention and treatment, and explored how gender roles influence awareness and acceptability of HPV vaccine. In anticipation of low prior knowledge of HPV vaccination, the researchers adapted the approach of Kelly-Hanku and colleagues (22). After a brief discussion to capture participants' existing understanding of the vaccine, the research team provided information and explained HPV vaccination. This was done before asking about acceptability of the HPV vaccine, to capture participants' thoughts and concerns regarding the impending introduction of the HPV vaccine in India.

The study was co-designed by co-authors, with data collection tools developed collaboratively. Fieldwork was conducted by PL, supervised by PR, a senior female public health researcher trained in qualitative research methods. Field investigators who spoke the local language were engaged in each site for data collection. All investigators were oriented on study objectives, interview guides, and ethics by PR & PL. Guides were translated, back-translated, and pilot-tested for quality. Field notes were taken during SSIs and FGDs to supplement data.

### 2.3 Data management and analysis

After each day, field notes were written by the research team and discussed with the field investigator for their observation inputs. Digital recordings of FGDs and interviews were kept in a separate folder accessible to the research team. Recordings were transcribed by professional transcribers, translated from local languages to English, and checked for consistency. All transcripts were then cleaned, and analysis was conducted using Nvivo 1.7.1 software.

The study used thematic analysis, following Braun and Clarke's method (57). All transcripts were read by UV to ensure familiarization with data, identify emerging themes, and create initial codes. A codebook was prepared based on initial data review, followed by iterative discussions with PR & PJ. All codes were brought together, and a codebook was created. Data were then systematically coded using both inductive and deductive approaches After finalizing the themes, categories and codes were created and applied across the dataset.

This paper adheres to the Consolidated Criteria for Reporting Qualitative Research (COREQ) checklist (58), addressing all relevant items.

### 2.4 Ethical considerations and informed consent

Each study participant had the study explained to them and were provided a written information sheet at which time all queries were addressed. Written signed consent was obtained by each participant to conduct and record the interview. In the case of a participant who could not sign, a thumb impression was used to provide consent, and was then signed by a witness. To ensure anonymity, a unique identification code was allocated for each participant, and names and other identifiable information was removed from transcripts.

Ethical approval for the conduct of this study was granted by The George Institute for Global Health India (Project Number 24/2021) and ratified by the Human Research Ethics Committee (HREC), UNSW Sydney. The study also received ethics approval from the National Institute of Cancer Prevention and Research (NICPR), (NICPR/IEC/2022/018) and Kasturba Medical College, Kasturba Hospital Institutional Ethics Committee (IEC1-21-2023). Permission was also obtained from the Integrated Tribal Development Project, Udupi, Karnataka. The study also received approval form the Health Ministry's Screening Committee (HMSC), Ministry of Health and Family Welfare, Government of India (Proposal ID: 2023-17647).

## 3 Results

### 3.1 Participant profile

A total of 146 women and 28 men participated in the study (Table 2). This included 89 women who participated in SSI, 34 of whom had ever screened and 55 who had never screened. The majority of women involved in SSI were aged between 30 and 50 years (70%, *n* = 63). Most participants were married (74%, *n* = 66) and over half resided in rural areas (56%, *n* = 50). Educational attainment levels of women were low with 25% (*n* = 22) reporting no schooling and 18% (*n* = 16) reporting only having primary education. About half, (51%, *n* = 45) identified their occupation as being a homemaker.

**Table 2:** Demographic characteristics of the study participants.

Characteristic	Semi-structured interviews, *n* (%) (*N* = 89)		Focus group discussions, *n* (%) (*N*_FGD_ = 12)^a^	
	Screened for cervical cancer (*N* = 34)	Not screened for cervical cancer (*N* = 55)	Women (*N* = 57)	Men (*N* = 28)
Sites				
Delhi	14 (41.2)	17 (30.9)	26 (45.6) (*N*_FGD_—4)	6 (21.4) (*N*_FGD_—1)
Karnataka	19 (56.9)	29 (52.7)	16 (28.1) (*N*_FGD_—2)	8 (28.6) (*N*_FGD_—1)
Tripura	1 (0.03)	9 (16.4)	15 (26.3) (*N*_FGD_—2)	14 (50) (*N*_FGD_—2)
Category				
Migrant	14 (41.2)	14 (25.5)	13 (22.8) (*N*_FGD_—2)	6 (21.4) (*N*_FGD_—1)
Tribal	11 (32.3)	10 (18.2)	9 (15.8) (*N*_FGD_—1)	8 (28.6) (*N*_FGD_—1)
Women living with HIV (WLHIV)	5 (14.7)	12 (21.8)	14 (24.6) (*N*_FGD_—2)	-
Female Sex worker (FSW)	3 (8.8)	10 (18.2)	6 (10.5) (*N*_FGD_—1)	-
Internally displaced people (IDP)	1 (2.9)	9 (16.4)	15 (26.3) (*N*_FGD_—2)	14 (50) (*N*_FGD_—2)
Age (in years)				
30–40	9 (26.5)	24 (43.6)	35 (61.4)	13 (46.4)
41–50	15 (44.1)	22 (40)	13 (22.8)	9 (32.1)
51–65	10 (29.4)	9 (16.4)	9 (15.8)	6 (21.4)
Residence				
Urban	17 (50)	22 (40)	35 (61.4)	6 (21.4)
Rural	17 (50)	33 (60)	22 (38.6)	22 (78.6)
Marital status				
Single	1 (2.9)	0 (0)	1 (1.7)	0 (0)
Married	26 (76.5)	40 (72.7)	44 (77.2)	28 (100)
Divorced	0 (0)	2 (3.6)	1 (1.7)	0 (0)
Widow	7 (20.6)	11 (20)	8 (14.0)	0 (0)
Separated	0 (0)	2 (3.6)	3 (5.3)	0 (0)
Education				
No schooling	6 (17.6)	16 (29.1)	15 (26.3)	8 (28.6)
Primary school	6 (17.6)	10 (18.2)	12 (21.0)	9 (32.1)
Middle school	6 (17.6)	8 (14.5)	5 (8.8)	5 (17.8)
Secondary school	4 (11.8)	14 (25.5)	17 (29.8)	3 (10.7)
Higher secondary	9 (26.5)	6 (10.9)	6 (10.5)	2 (7.1)
Graduate and above	3 (8.8)	1 (1.8)	2 (3.5)	1 (3.6)
Employment/Occupation				
Homemaker	14 (41.2)	31 (56.4)	32 (56.1)	0 (0)
Self-employed	3 (8.8)	1 (1.8)	0 (0)	2 (7.1)
Government employee	1 (2.9)	0 (0)	0 (0)	7 (25)
Private employee	6 (17.6)	8 (14.5)	11 (19.3)	6 (21.4)
Sex worker	3 (8.8)	10 (18.2)	6 (10.5)	0 (0)
Retired employee	0 (0)	1 (1.8)	0 (0)	0 (0)
Others^b^	7 (20.6)	4 (7.3)	8 (14.0)	5 (17.8)

^a^ *N*_FGD_—Number of FGDs.

^b^ Occupation-others include domestic worker/house-helps, Community Health worker and daily wage labourer.

A total of 57 women and 28 men participated in 12 gender-specific FGD (8 among women and 4 among men). Most women in FGDs were aged 30–50 years (84%, *n* = 48) and reported being married (77%, *n* = 44), with 61% (*n* = 35) living in urban areas. The majority of women self-reported their occupation as a homemaker (56%, *n* = 32). All men were married and 79% (*n* = 22) resided in rural areas. Most men were employed in the government (25%, *n* = 7) or private sector (21%, *n* = 6). Equal proportions of both women and men in FGDs had no formal education (women-26% and men-29%).

In the sections below, we present key findings on awareness of the HPV vaccine, perceptions of the ideal age for vaccination, attitudes toward vaccination, decision-making autonomy, major barriers and facilitators to uptake, and participants' practical suggestions for improving coverage.

### 3.2 Knowledge about HPV vaccination

The majority of participants across the three sites had not heard about HPV vaccination and were unaware of its purpose. Only four participants demonstrated knowledge about the vaccine and its implications. As one sex worker from Delhi stated, “*I have heard about the name of the vaccine, but I don't know much*.” Those who had heard about the vaccine mentioned medical camps or NGO awareness campaigns as their source of information.

“When we had been to a [medical] camp, I vaguely remember that they had said that it [HPV vaccine] is there”. (SSI, Tribal woman, Karnataka)

Concurrently, knowledge about HPV as a virus, its transmission, and the need for vaccination was low among participants. Some participants confused the effect of vaccination with treatment, describing the HPV vaccine as a therapeutic intervention. For example, a migrant man in Delhi believed the HPV vaccine was given to young girls to prevent vaginal discharge (white discharge/leucorrhoea), rather than cervical cancer. Misconceptions about the vaccine's purpose and dosage were common among participants.

“There is a vaccination which has come. If that vaccination is given before the puberty to the girl, then she will not have the problem of white discharge” FGD (Men), Migrant, Delhi.

After being informed about HPV vaccination, participants were asked about their views on the ideal age for vaccination. Most participants were unclear about the ideal age for HPV vaccination. Those who suggested an age recommended vaccinating girls in their teenage years (13–17 years), before sexual activity begins. Some women in Delhi and Karnataka suggested vaccination for older women (30–45 years), with one tribal woman from Karnataka proposing vaccination after 40, believing women are protected from cancer during reproductive years. Views varied, with most suggestions coming from women, primarily in Delhi.

“If the girl is 13 years old and she has not had sex even once. Only then she can be given the vaccine. If she has had a physical relationship, then she will not be given the vaccine nor will it work on her.” SSI, PLHIV, Delhi

#### 3.2.1 Acceptance and attitudes towards HPV vaccination

Over half of participants expressed positive attitudes towards the HPV vaccine, reporting willingness to receive it themselves or have their daughters vaccinated. They saw the vaccine as invaluable for preventing cervical cancer and ensuring a better future for their daughters. As one female sex worker from Karnataka said, “*Yes I would like to get her [daughter] the vaccine because she would not have to face the problem [cervical cancer] in future, she can live well, nothing can be happier than that*.” A woman with HIV from Delhi recognized her high risk of cervical cancer and wanted vaccination.

Some participants drew on their COVID-19 experience to support HPV vaccination, citing trust in government-led vaccination efforts.

“The way it was done for coronavirus, the same way, we [will] all agree with this as well.” (FGD, Migrant woman, Delhi).

Concurrently, a few participants expressed concerns about potential side effects, citing COVID-19 vaccine experiences (numbness, dizziness, weakness). Some participants also expressed that they were worried about fertility impacts on young girls. One woman suggested trying the vaccine on herself first: “*If this vaccine is safe then I will first try it on myself and then give it to my daughter.*” (SSI, Migrant Women, Delhi). Some participants also noted that they would only consider vaccination if others in their community did so too.

An IDP participant from Tripura highlighted the importance of tribal language messaging:

“In our bru [tribal language], if I explain to them, one or two [persons] will understand and get vaccinated. But others might say that if there's an infection or if someone falls ill, will you take responsibility for it? But there are also many people who understand that if explained properly, they might agree.” (SSI, Internally Displaced, Tripura)

A few male participants from Tripura also shared that members of the community, especially women, reject vaccination due to fear. In contrast to those who expressed that the vaccine drive associated with COVID-19 would support HPV vaccination, others were concerned that the fear in the community about vaccination is so widespread that as an internally displaced man living in Tripura said, “*People run away just by hearing the word vaccine*.” He went on to say that such fear only exists for vaccinations that require an injection with a needle, but not so much with oral vaccines.

Some participants saw the vaccine as unnecessary if women lived “moral lives” with few sexual partners.

“If we live properly, I don't think there's a need to get vaccinated.” (SSI, Internally Displaced Woman, Tripura).

#### 3.2.2 Decision making for HPV vaccination: a gendered perspective

Like many family health decisions, gender plays an important role in vaccination uptake. Regarding HPV vaccination, the decision-making authority in the family varied across the groups with some important regional and gendered differences.

Most women in Delhi who participated in SSI claimed autonomy stating that they could decide themselves to get vaccinated and vaccinate their daughters.

“I will take the decision [to vaccinate]. I will still go because this is regarding my health take it.” (SSI, Migrant woman, Delhi.)

In contrast, fewer women in Karnataka and none in Tripura saw themselves as primary decision-makers for any future HPV vaccine. About half the women across sites sought their husband's permission, with migrant women in Delhi emphasizing this. About half of women in Karnataka and three each from Delhi and Tripura reported joint decision-making with husbands. Delhi FGDs revealed husbands typically decided on vaccinating daughters. Others, including men in Delhi and Tripura, shared decision-making included extended family members like husband's parents.

“The male will take the decision [for HPV vaccination]… See, we [women] have men above us, so we have to take opinions from them as well.” (FGD, Women, Migrant, Delhi).

A few women from Karnataka said that health care professionals, including nurses and doctors, were important decision makers in deciding if a women or girl is vaccinated.

“Either it is the mother, or the sisters [nurses] or doctors will take the decision [to vaccinate against HPV].” SSI, Sex Worker, Karnataka.

#### 3.2.3 Suggestions for implementing HPV vaccination in the community

To ensure a successful HPV vaccine program in India, participants identified several key strategies. Community-based initiatives were emphasized, including door-to-door campaigns, medical camps (especially for displaced people), engaging women's groups and community workers, and community-level awareness programs in local languages using social media platforms (Facebook and WhatsApp), other media platforms, and edutainment through street performances.

Some participants also advocated for making HPV vaccination mandatory for girls and women, drawing on the COVID-19 experience. As one female sex worker from Delhi said, “*[Government should] set up tents and camps and make announcements that this [HPV] vaccine will prevent cancer… If they will set up camps and tents, then automatically people will come like when people got to know about Covid vaccine they went there and got themselves vaccinated*”.

## 4 Discussion

This study explored the knowledge, attitudes, and acceptability surrounding HPV vaccination among marginalized communities across three diverse Indian contexts. Recent evidence from systematic review and meta-analysis from India already reported low levels of knowledge, attitude and HPV vaccination coverage in India (59). Our findings reveal a critical gap in awareness about the HPV vaccine, with only a small proportion of participants, mainly HIV-positive women, demonstrating knowledge of HPV vaccination. Notably, there was almost no awareness of the HPV vaccine among male participants, with only one participant having heard of the vaccine. This reflects a significant gender disparity in awareness, which is particularly concerning given that men often act as key decision-makers in familial health matters. This aligns with earlier studies in India (29, 60, 61) and other low- and middle-income countries (LMICs) (62–65) that consistently report low awareness levels about HPV vaccination, particularly among marginalized populations (66–69), and among men (70–72).

Participants' perceptions regarding the appropriate age for vaccination were mixed. While some correctly identified the reasoning that girls should be vaccinated before they become sexually active, the specific age pointed out was 13–17 years which is later than the 9–14 years as specified in global (73) and national guidelines (74). Other participants suggested later ages, even up to 45 years. Such beliefs appear to stem from misconceptions about the purpose and mechanism of the vaccine, particularly the notion that it is a therapeutic rather than preventive intervention (75).

Attitudes toward HPV vaccination ranged from supportive to cautious and, in a few cases, negative. Encouragingly, a majority expressed willingness to accept the vaccine, often drawing parallels with the COVID-19 vaccination campaign. Trust in governmental health initiatives and prior experience with large-scale immunization appeared to positively influence their receptivity. This was also demonstrated in recent literature in the context of COVID-19 vaccination (76–78). However, concerns about potential side effects (numbness, dizziness, weakness, and fertility issues), informed by anecdotal experiences with COVID-19 vaccines, were common. Such apprehensions have been documented in other LMIC settings (79–82) and highlight the need for transparent communication about HPV vaccine safety and side effects for its acceptance (83, 84).

A small subset of participants moralized the vaccine, and by association, the infection itself. They suggested that “proper” conduct negates the need for vaccination. These views reflect socio-cultural beliefs and stigma around female sexuality in India. They echo previous research that highlights concerns of female sexual morality demonstrating how moral reasoning negatively influence vaccine acceptance (85–87).

Decision-making autonomy was another key theme. While some women, especially in Delhi, reported making independent decisions about their health and that of their daughters, others-particularly in Tripura and parts of Karnataka-described a more patriarchal structure where husbands and their parents, or elder members of the family were those with the authority to make important health decisions associated with HPV vaccination. These findings reflect gendered power dynamics in health-related decision-making as elsewhere in similar patriarchal societies (88–90).

Participants offered practical suggestions for community-based HPV vaccination implementation, including door-to-door outreach, engagement with local health workers, and leveraging media platforms and women's groups. The community-driven suggestions offer locally relevant solutions that can help bridge the knowledge gap and make vaccination efforts more effective and acceptable. Involving communities in planning not only builds trust but also ensures that strategies address real barriers like low awareness, mistrust, and limited access.

As cautioned by others prior to the role out of the HPV vaccine elsewhere (22) a failure to attend to the contexts and local milieus, and gendered power dynamics where the HPV vaccine is being implemented, may result in a failure to engage communities, or worse result in outright rejection and opposition. India can ill afford to this amongst those already marginalized.

Our study highlights that HPV vaccine uptake is shaped by a complex interplay of individual, socio-cultural, and health system factors. Key barriers include low awareness about the HPV vaccine and the recommended age for vaccination, uncertainty regarding the benefits of vaccinating older women, and deeply rooted cultural beliefs linking cancer risk to reproductive years. Concerns about stigma, particularly the perception that vaccinating adolescent girls implies sexual activity, further hinder acceptance. At the same time, several facilitators offer important opportunities to improve uptake, including the recognition of the benefits of vaccinating girls before the onset of sexual activity, strong community trust in government-led immunization programs, and the positive impact of targeted awareness and education initiatives. Addressing cultural beliefs and misconceptions about cancer risk also emerged as critical. Together, these findings point towards the need for health systems to adopt context-sensitive strategies that enhance awareness, leverage existing trust in public programs, and proactively engage with socio-cultural concerns to ensure equitable and effective implementation of HPV vaccination.

### 4.1 Recommendations

Based on these findings, we recommend a set of policy and program strategies aimed at increasing HPV vaccine acceptance and ensuring equitable access for marginalized groups. Key actions include: addressing structural, informational, and social barriers that influence vaccine decision-making, and adopting implementation approaches that are culturally appropriate, community-informed, and responsive to the specific needs of marginalized populations. At the same time, we suggest that future research explicitly focus on identifying and closing the information gaps that currently limit uptake among these populations.

#### 4.1.1 Policy and program implementation

While planning the rollout of HPV vaccination, particularly in marginalized communities, the Government of India must address the widespread lack of awareness about HPV and its causative role in cervical cancer. Policies should ensure that basic, culturally grounded education is delivered in local languages and through trusted community actors. Such communication strategies should be explicitly designed to be culturally appropriate and context-sensitive, addressing locally prevalent myths related to fertility, vaccine safety, and moral concerns in a non-judgmental manner. Information must clearly explain that the vaccine is preventive (not therapeutic), emphasize the recommended age for vaccination, and openly discuss safety concerns. Misconceptions tied to sexuality and moral behavior also need to be addressed without judgement to ensure that adolescent girls can access the vaccine without stigma. Given the strong influence of men and other family members in health decision-making, especially in patriarchal settings, national vaccination strategies should explicitly include outreach to men. Targeted engagement of fathers and key male decision-makers through community-based platforms can support informed and supportive household decisions. Healthcare provider sensitization and capacity building to reduce stigma and discrimination are also essential for making HPV vaccination acceptable and accessible among PLHIV and FSW (91).

Furthermore, practical, community-based, and community-driven strategies should guide HPV vaccine implementation. Insights from participants in this study can inform an inclusive and responsive plan for reaching marginalized and hard-to-reach groups. In contexts with high numbers of girls out of school and limited formal education, school-based programmes may be insufficient.

Evidence from Rwanda, South Africa, and Bhutan (92–95) indicates that school-based delivery paired with “mop-up” programmes targeting out-of-school girls, and supported by strong community awareness efforts, yields the best results. If India adopts school-based vaccination, ensuring structured pathways for out-of-school girls will be essential. Engagement of trusted intermediaries such as frontline health workers, teachers, peer educators, and local leaders can enhance outreach, credibility, and uptake. Grounding programmes in the lived realities of each community will be key to building trust and improving vaccine access among marginalized groups (96).

HPV vaccination programmes should strengthen accountability, transparency, communication, and culturally appropriate engagement within the health system, particularly for marginalized communities. Evidence from other settings indicates that concerns about information quality, side effects, provider characteristics, and broader social insecurity shape HPV vaccine perceptions (20, 97, 98). Addressing these factors alongside vaccine delivery may improve uptake.

We recommend integrating HPV vaccination within existing HIV care services, with coordination from institutions such as the National AIDS Control Organization (NACO), as an effective strategy to expand coverage among marginalized groups, especially PLHIV and female sex workers. Evidence from low-resource settings shows that such integrative approaches, delivered through adolescent health programmes, can be particularly effective (96). Leveraging the existing school health program platform could further facilitate HPV vaccination among adolescent girls. Integrating the HPV vaccine into school-based health services would enable outreach to girls in their teenage years (13–17 years), help overcome logistical barriers such as access and awareness, build on the trust already placed in school health systems, and provide an effective channel for targeted education and awareness initiatives.

#### 4.1.2 Recommendations for research

Further research should examine implementation models that strengthen trust, improve accountability, and sustain engagement in marginalized settings. Such studies can explore how different approaches can help address entrenched mistrust of the health system. In-depth research is also needed to understand how contextual factors like displacement, social insecurity, and gendered power dynamics shape acceptance of HPV vaccination. Generating this evidence will be essential for designing implementation strategies that are responsive, equitable, and effective for the communities most at risk.

### 4.2 Limitations and strengths of the study

The study may be subject to sampling bias due to the use of purposive and snowball sampling, limiting the representativeness of the findings. Additionally, participants' views on HPV vaccination may have been influenced by the information provided during the interviews, potentially shaping their responses. These factors should be considered when interpreting the findings. Additionally, as a qualitative study, these findings are neither generalizable across India, nor of all marginalized communities. These findings are shaped by the specific sociocultural and geographic contexts of the study settings, which may limit their transferability to other regions or populations. Despite these limitations, this study provides important insights as, to our knowledge, it is the first to examine HPV vaccination among marginalized communities in India, thereby contributing valuable evidence at a key moment in India's efforts to eliminate cervical cancer as a public health issue.

## 5 Conclusion

This study shows that as India undertakes efforts to eliminate cervical cancer as a public health issue, and roll out HPV vaccination, both women and men from these communities must be prioritized. But above this, their fears and concerns, gender and familial dynamics around decision making, and strategies for engagement and health information sharing, as illustrated here, need to be attended to.

## Data Availability

The raw data supporting the conclusions of this article will be made available by the authors, without undue reservation.
